# Age-Based Positivity Effects in Imagining and Recalling Future Positive and Negative Autobiographical Events

**DOI:** 10.3389/fpsyg.2017.01700

**Published:** 2017-09-27

**Authors:** Elvira García-Bajos, Malen Migueles, Alaitz Aizpurua

**Affiliations:** Faculty of Psychology, University of the Basque Country UPV/EHU, San Sebastián, Spain

**Keywords:** autobiographical memory, future events, positive and negative experiences, positivity effect, young and older adults

## Abstract

Thoughts about the future reflect personal goals, and projections into the future enrich our emotional life. Researchers have taken an interest in determining whether the tendency to remember more positive than negative emotional events observed when recalling past events also appears when remembering imagined future events. The objective of this study was to examine the age-based positivity effect of recall for future positive and negative autobiographical events in young and older adults. Representative future events were first established to develop the cues used to prompt personal future events. In the production task, the participants were presented with eight positive and eight negative random future events of young or older adults as a model and the corresponding cues to generate their own positive and negative future autobiographical events. In the recall task, the participants recovered as many experiences as they could of the model and the positive and negative events produced by themselves. The participants correctly recalled more positive than negative events and committed more errors for negative than positive events, showing a clear tendency in both young and older adults to recall future imagined events as positive. Regarding age, the young adults recalled more events than the older participants whilst the older participants in particular showed better recall of their own imagined future events than the model’s events, and committed more errors when recalling the model’s events than their own imagined events. Regarding the positivity effect in incorrect recall, more than half of the errors were valence changes, most of these being from negative to positive events, and these valence changes were more pronounced in the older than in the younger adults. In general, there were fewer differences between young and older adults in the recall of positive events in comparison with negative events. Our findings suggest that people are well disposed toward recalling positive imagined future events and preserve a positive emotional state, suppressing negative memories.

## Introduction

Episodic memory not only includes past experiences, but is also the vehicle that allows us to travel mentally through time from the past and into the future (Tulving, 1985). Thinking about past experiences and imagining the future are common occurrences in our daily thoughts, conversations, and social relations. In recent years both cognitive psychology and the neurosciences have taken an interest in understanding whether the projection of oneself—the episodic thoughts of future experiences—shares characteristics with the episodic recall of past events (for reviews, see Schacter et al., 2007; Szpunar, 2010). Finnbogadóttir and Berntsen (2013) investigated the frequency and valence of spontaneously arising experiences and they found that involuntary future projections were as frequent as the involuntary retrieval of past events. They observed that more positive events were reported than negative experiences (see also Berntsen and Rubin, 2002; Newby-Clark and Ross, 2003; Berntsen and Jacobsen, 2008), and that this preference for positive events was greater for future than for past events. It has also been observed that older adults tend to remember positively the past (Kennedy et al., 2004), and subjectively rate past and imagined future events more positively than younger adults (Gallo et al., 2011).

Although the majority of past experiences and future thoughts include trivial and unemotional events (e.g., thinking about the day’s activities or reviewing the shopping list), positive and negative emotional experiences are what give meaning to our lives and they provide us with our own identity (Conway and Pleydell-Pearce, 2000; García-Bajos and Migueles, 2013; Demblon and D’Argembeau, 2017). Thinking about future events can be as relevant to present life as evoking past autobiographical experiences. In fact, to recreate the future we use our knowledge and past experiences. Mental simulations of future experiences are often concerned with emotionally arousing events and virtually nothing is known about memory for these simulations or the impact of their emotional valence on thoughts about the future in young and older adults. The aim of this study, therefore, was to examine whether there are differences in the recall of future positive and negative autobiographical events between young and older adults. That is, to examine age-based positivity effects in imagining and recalling imagined future events.

The term positivity refers to the preference for positive information as opposed to negative content in attention and memory tasks, an effect that is accentuated with age (Carstensen and Mikels, 2005; Mather and Carstensen, 2005; Schryer and Ross, 2014). The positivity effect concerns the relative difference between older and younger people in attention to and memory for positive as opposed to negative material (see Reed and Carstensen, 2012). Young adults show enhanced memory for both positive and negative information, and often have a tendency to process and remember negative information more thoroughly than positive information (for a discussion, see Baumeister et al., 2001). Compared with young adults, older people remember more positive than negative content, or show reduced memory for negative information when attending to visual materials such as scenes, affective pictures, or faces (Charles et al., 2003; Reed et al., 2014; Mammarella et al., 2016). For instance, it has been observed that older people spend more time attending to positive faces that reflect feelings of joy than negative faces that express anger or sadness (Mather and Carstensen, 2003). This positivity effect has also been observed in memory for word lists (Shamaskin et al., 2010), long-term autobiographical experiences (Kennedy et al., 2004), or in tasks that involve working memory (Mikels et al., 2005). Gallo et al. (2011) even found that older adults subjectively rated retrieved autobiographical past events or imagined future events more positively than younger adults, demonstrating an age-related positivity effect. However, the opposite pattern of results has also been found (e.g., Grühn et al., 2005), or in some cases negligible differences have been observed between young and older adults in the processing of emotional information (for a review see Reed and Carstensen, 2012). The variability in both the size and nature of the positivity effect in remembering suggest that there are factors that have received little attention that could moderate the effect, such as encoding conditions and information content (Hess et al., 2013). Reed et al. (2014) conducted a systematic meta-analysis of 100 empirical studies of the positivity effect. All the studies compared positive and negative emotional material in young and older adults, which allowed for an analysis of the interaction between emotional valence and aging. The results indicated that the positivity effect is clear and consistent, particularly when processing constraints are not imposed on participants and natural information is processed.

An important factor in the memory of positive and negative emotional experiences is self-referencing (e.g., Gutchess et al., 2007; Leshikar et al., 2015). Therefore, in this study we examined the memory of autobiographical future emotional events related to the participants themselves and those of other people that, in addition, share (or not) age with the participants of the experiment. Recovering, generating, or producing self events require deeper and more elaborate processing than when simply reading about the experiences of others (Symons and Johnson, 1997), and both young and older adults can benefit of depth of processing (Lalanne et al., 2013). Therefore, personal events will be remembered better than those of other people. In addition, this more elaborate processing may modulate the positivity effect in recalling imagined future events. It has been shown that both young and older adults can benefit from self-referenced items (Gutchess et al., 2007; Lalanne et al., 2013), that self-referencing does not restore the memory of older adults to the level of young adults (Gutchess et al., 2007), and that self-reference effect can be more marginal for older adults (Lalanne et al., 2013). Furthermore, inconsistent with a positivity effect in aging, Leshikar et al. (2015) found that self-referencing increased recollection memory for positive items in both young and older adults, but further study is needed in this area. We also examined the impact of a possible identification of the participants with the experiences of the reference model. Thus, personal information or the experiences of people who share age, life scripts, life cycle, concerns and illusions, may receive additional attentional resources. In other words, self-referential processing, which involves encoding information in relation to oneself, can improve the memory of the future events of other people (Gutchess et al., 2007; Leshikar et al., 2015) and also modulate the positivity effect. Therefore, additional objectives of the present study were to examine the impact of self-reference and identification with other people’s experiences in the positivity effect.

Why do older people show a tendency to favor positive information over negative information? Although the underlying cognitive mechanisms of the positivity effect are not fully understood, several alternatives have been considered in the search for explanations. The *Socio-emotional Selectivity Theory* (SST; Carstensen, 2006) emphasizes an age-related increase in the accessibility of positive information. This theory posits that with increased age, the priorities and motivations of a person shift according to their future prospects. The fact that future prospects become narrow over time and the fragility of life is appreciated leads to a prioritization of current objectives that are related to self-satisfaction. In other words, older adults deploy cognitive control mechanisms to avoid negative information and to seek positive, emotionally rewarding information. Prioritizing the positive means that when it comes to coding information, paying attention, or remembering facts, there is a preference for what is deemed to be pleasant and positive. Other theories emphasize cognitive mechanisms (e.g., Spaniol et al., 2008) and neural processes (Kensinger and Schacter, 2008; Mammarella et al., 2017) to explain the age × valence interaction. Studies of young adults have shown that inhibitory mechanisms may help keep negative thoughts and episodes from coming to mind, promoting a positive bias (e.g., Giebl et al., 2016; García-Bajos and Migueles, 2017), and studies with young and older adults reveal that older adults recruit cognitive control processes to strengthen positive and diminish negative information (e.g., Mather and Knight, 2005; Knight et al., 2007), or older people automatically prefer and process positive information because it is less complex than negative content (Labouvie-Vief et al., 2010; Wurm, 2011). Szpunar et al. (2012) suggest that one basis of the preference for positive information may be the *fading affect bias* (Walker et al., 1997), whereby information related to negative emotions tends to fade more rapidly than that related to positive emotions, which results in a tendency to remember the positive. Consistent with this interpretation, Berntsen et al. (2011) observed that older people remembered and judged positive experiences as being more central to their life and identity than negative or traumatic experiences.

Although errors have been analyzed in the positivity effect, showing that older participants show an optimism bias and recall more false positive than false negative information (e.g., Fernandes et al., 2008), one aspect that is rarely studied in the phenomenon of positivity effect in memory is the nature of errors. The majority of the studies have focused on the production of events, the description of experiences, phenomenological evaluations, or correct recall (Reed et al., 2014). The properties of memories and the types of memory errors people commit offer a window into the organization of memory (Schacter, 1999). Could signs of this bias in favor of positive over negative information in later life also be detected in memory errors? It has been found that older adults are more prone than younger adults to make everyday memory errors (Ossher et al., 2013; Devitt and Schacter, 2016). The limitations of the elderly appear in the memory of information that requires attentional and cognitive resources (Craik and McDowd, 1987; Danckert and Craik, 2013), such as specific facts or concrete details. In addition, future thinking in older adults is characterized by a lack of specificity of imagined events and by an equal or even higher subjective experience in comparison with younger adults (Jumentier et al., 2017). However, it has been observed that age differences in memory are reduced or even eliminated when participants process emotional or affective information (May et al., 2005). Thus, an additional goal of this study was to examine the nature of the errors shown in young and old adults, to determine whether their impact is greater on positive or negative content, and to also identify the types of errors that are made in the recall of imagined future events. For this, we distinguished between commission errors, source errors, and emotional valence changes. Commission errors, when the participants contribute their own or others’ events that are not present in the coding phase, may be connected to prior knowledge (Migueles and García-Bajos, 2012), life scripts (Rubin and Berntsen, 2003), or forms of semantic memory that can be used to guide one’s anticipated future (Grysman et al., 2015). Source errors—in which an error is made regarding the subject or action of an event—can be an index of the lack of specificity of processing the origin of the information (Danckert and Craik, 2013; Jumentier et al., 2017) and should depend on the recollection of specific details about the earlier generated events (Gallo et al., 2011). And more relevant to the effect of positivity in the memory of future events would be changes in emotional valence, where negative events are remembered as positive.

In short, in our daily lives we frequently think of future positive and negative emotional events. Because of the interest in understanding the effects of aging on episodic future memory, in this study we examined the positivity effect and differences in recall between young and older adults for self and other future events. We also examined errors, since they are indicators of memory processes and limitations, and may be manifest in positivity or negativity biases in the recall of emotional future events.

## Materials and Methods

### Participants

The final sample of participants in the experiment consisted of a total of 136 students from the University of the Basque Country. Of these, there were 68 young adults aged between18 and 30 years (*M* = 20.37; *SD* = 2.16), which included 59 women and 9 men, all of which were psychology students. The 68 older participants were aged between 55 and 75 years (*M* = 65.13; *SD* = 4.02), 48 of which were females and 20 males. These old participants were enrolled in a university degree in human sciences. **Table 1** displays the characteristics of the participants. There were no differences between the young and older adults in the years of completed formal studies, *t*(134) = -94, *p* = 0.35, *d* = 0.02, or perceived health, Mann–Whitney test, *Z* = -1.84, *p* = 0.07. Young adults showed greater semantic fluency in the animal production task for 1 min, *t*(134) = 5.79, *p* < 0.001, *d* = 0.21, and greater processing speed in the Wechsler Adult’s digit symbol-coding task Intelligence Scale (WAIS, Wechsler, 1997/1999) for 2 min, *t*(134) = 10.33, *p* < 0.001, *d* = 0.36, in comparison with the older adults. In contrast, the older participants achieved higher scores than the young adults, *t*(134) = 2.65, *p* < 0.01, *d* = 0.17, in a 4-min verbal comprehension task composed of 50 items, each of which included four synonyms (Thurstone, 1938/1996). To examine whether the significant differences in cognitive abilities between the young and older participants influenced the main findings of the study on future events production, correct recall or errors, a set of ANCOVAs (Analyses of Covariance), were conducted with semantic fluency, processing speed and verbal comprehension as covariables. The results showed that semantic fluency, processing speed or verbal comprehension did not interact significantly (*p* > 0.05 cases) with any of the factors studied: age group, model age, emotional valence, experiences recalled or type of errors.

**Table 1:** Participant characteristics (*SDs* in parentheses).

	Age	Years of education	Health^a^	Semantic fluency^b^	Verbal comprehension^c^	Speed of processing^d^
Young	20.37 (2.16)	15.50 (0.82)	4.32 (0.58)	18.18 (2.75)	34.12 (6.50)	86.35 (14.25)
Older	65.13 (4.02)	15.85 (2.98)	4.15 (0.55	14.71 (4.11)	38.85 (7.69)	59.88 (15.61)

^a^*Participants rated their state of health on a scale from 1 (very bad) to 5 (very good). ^b^1 min of animal names. ^c^PMA Verbal factor (Thurstone, 1938/1996).*

^d^*Digit symbol-coding task (Wechsler, 1997/1999)*.

### Materials

For the experiment, each participant was presented with 16 future experiences, eight positive and eight negative (**Table 2**) randomly organized. To manipulate the model age (young or old) as a between-participants factor to generate self-events, the 68 young adults and the 68 older participants were randomly divided in two subgroups of 34 participants each. In addition, two examples were selected to control for primacy and recency effects, which also served to help participants understand the task. Future experiences had been obtained from a previous normative study using 600 participants of similar characteristics to those of the current study, but none of them subsequently participated in the present experiment. This sample was composed of 300 youngsters aged between 18 and 30 years (*M* = 20.46, *SD* = 2.38), of which 243 were females and 57 males. The 300 older participants were aged between 56 and 80 years (*M* = 66.59, *SD* = 5.09), of which 212 were females and 88 males. All participants produced, for 8 min, future events. Of these, 100 young and older participants produced positive thoughts or experiences, 100 produced negative thoughts, and the remaining 100 did not receive instructions about the valence. Based on the experiences obtained, eight frequent positive and eight negative events were selected, generated by more than 20% of young or older adults. The 16 future events selected served as models for the participants in the experiment to generate their own future events or thoughts. Half of the young participants received young model events, whereas the other half received old model events, the same being true for older participants. Whether the model came from young or old adults, retrieval cues (a procedure based on Migueles and García-Bajos, 2015 and García-Bajos and Migueles, 2017) were selected to help participants produce their own future events or experiences, that could be positive (e.g., Travel to...) and negative (e.g., Fear of...).

**Table 2:** The 16 future autobiographical events, eight positive and eight negative, of the young and old model age (in italics the older events, when they were different from the young model events).

Future autobiographical events
**POSITIVE**
*Example*. **Attend** a concert/*play*
**Do** a master’s degree/*computing course*
**Travel to** Paris*/Italy*
**Having a good time** at parties
**Meet** my future partner/*my children’s partner*
**Have** children/*grandchildren*
**Get along** with family
**Buy myself** a house/*another house*
**Live** by myself/*independently*
**NEGATIVE**
**Not being able** to finish the degree/*finish the university courses*
**Fear of** not finding a job/*my children not finding a job*
**Contracting** a serious illness
**Arguing with** my friends
**Losing** my job/*cognitive abilities*
**Disappoint** my parents/*children*
**Death of** people close to me
**Suffer** an accident
*Example.* **Having problems** financially/*reaching the end of the month*

*Half of the young participants received young model events, whereas the other half received old model events, the same being true for older participants. The retrieval cues for the task of production of own experiences are in bold characters*.

### Design

The present study employed a 2 (participants age: young or old) × 2 (model age to generate self-events: young or old) × 2 (emotional valence: positive and negative), mixed factorial design with between-participants factors being the age of the participants and the age of the model to generate one’s own future experiences, whilst the within-participants variable was the emotional valence of the events. The correct recall and errors were measured for the recall of the positive and negative experiences of the model and those generated personally by each participant. Three types of errors were evaluated: commission errors, source errors, and valence changes.

### Procedure

Before starting the experiment, written informed consent was obtained from all participants. This study was carried out in accordance with the American Psychological Association standards for ethical treatment of participants, the Declaration of Helsinki, and was approved by the Ethics Committee of the University of the Basque Country UPV/EHU. Participants were first informed that the experiment dealt with the memory of positive and negative autobiographical experiences that are expected to occur in the future. They then filled out a personal information sheet, which included age, gender, education, and health status.

The experiment was conducted in three phases. The first phase focused on the task of producing future autobiographical experiences, followed by the second phase that included tests to measure participants’ cognitive abilities and, finally, the third phase consisted of the final recall task. The duration of the experiment was approximately 45 min.

To obtain the autobiographical experiences of the participants, a sheet was designed with the instructions in the heading and with two columns. The instructions were read by the experimenter and by each participant when receiving the material. The instructions were:

“When we think of the future we imagine positive and negative experiences that can occur in the near or distant future. We asked young and older university students to list their future thoughts and would like to contrast them with yours. Write your own positive or negative thoughts.”

The first column contained the 16 experiences obtained in the previous study with young psychology students or elderly human sciences students. The experiences of young and old served to counterbalance the model age so that the participants, young and old, generated their own experiences. At the head of the first column of the model experiences a 5 × 2.5 cm color photograph was placed with young or older students with headings *Psychology Students* or *Human Sciences Students* at the top of the photograph. To match the characteristics of the participants of the experiment, both photographs included white western female and male students in academic contexts; one photograph (young adults) representing undergraduate students in their twenties and the other photograph (old adults) elderly students in their sixties to seventies. The second column heading was an orange picture of a silhouette of the bust of a person on with the title *Me*. Below was a list with the cues of the 16 experiences, eight positive and eight negative (**Table 2**) presented randomly, but in the same order as the experiences of the model. The participants of the experiment, young and old, had in the first column the experiences of the model (young students or older students) and had to write in the second column their own future experiences. An example was included at the beginning and end of the lists in each column. Participants had 8 min to examine the experiences of the model, young or old, and use the cues to complete their own experiences. Therefore they had to generate 16 own experiences, eight positive and eight negative. They were told to work at their own pace and to try to complete all the cues.

Between the production task and the final recall three cognitive tests were intercalated. These also served as distractor tasks to fill the 15-min interval between the production tasks and the final recall task. The participants completed a 1-min verbal fluency test on animals, followed by a 4-min verbal comprehension test composed of 50 items containing 4-synonym groups, and finally a 2-min digit-symbol-coding task.

For the final recall task, the participants—young and old—were given a sheet with two blank columns, headed only by the same photos included in the production phase. They were told that they had 8 min to write in the order they wished, both the 16 future experiences of the model, young or old, and the 16 experiences of their own.

## Results

In the production task, it was taken into account that the participants had completed at least 14 of the 16 experiences. Those participants who had not correctly remembered at least two experiences of the model and two self experiences were also discarded. Two researchers independently evaluated the participants’ responses to the production and recall tasks, and there were no discrepancies between them.

### Production of Future Events

The success rate for the production of the 16 future events, eight positive and eight negative, was 98.91%. To analyze the events produced by the participants, a 2 (group age: young, older) × 2 (model age: young or older) × 2 (emotional valence: positive, negative) ANOVA was conducted. The factors model age, *F*(1,132) = 1.11, *p* = 0.74, $\eta_{p}^{2}$ = 0.001, and emotional valence, *F*(1,132) = 0.48, *p* = 0.49, $\eta_{p}^{2}$ = 0.004, were not significant, whereas the group factor, *F*(1,132) = 6.98, *p* = 0.009, $\eta_{p}^{2}$ = 0.05, and the interaction group × valence, *F*(1,132) = 4.36, *p* = 0.039, $\eta_{p}^{2}$ = 0.03, were significant. The number of events produced was higher in the young (*M* = 15.94, *SD* = 0.29, range 14–16) than in the older participants (*M* = 15.71, *SD* = 0.67, range 14–16). In the *post hoc* comparisons conducted to explore the group × valence interaction, the Bonferroni test revealed that there were no differences between the production of positive and negative experiences in young people (7.94, 8.00, *p* > 0.05) or in the elderly (7.91, 7.79; *p* > 0.05). In the production of positive experiences no differences were found between the young and older participants (7.94, 7.91; *p* > 0.05), but the older adults produced fewer negative experiences than the younger ones (7.79 < 8.00, *p* = 0.03). Therefore, although older people produced fewer experiences than young people, this limitation only affected negative, but not positive experiences. Thus, although the effect size was small, we found a positivity effect in the elderly in the task of producing self future events.

### Correct Recall

Of the 16 experiences of the model or of their own that the participants could remember, the young people correctly remembered between 3 and 15 (*M* = 9.01, *SD* = 2.43) experiences and the older people between 2 and 12 (*M* = 6.32, *SD* = 2.09) experiences. The percentage of experiences correctly recalled was greater than 50% in the recall of one’s own experiences (*M* = 53%, *SD* = 16.35), *t*(135) = 2.00, *p* < 0.05, *d* = 0.17, and less than 50% in the recall of the model experiences (*M* = 43%, *SD* = 16.56), *t*(135) = -4.92, *p* = 0.001, *d* = 0.42. **Table 3** displays the proportion of positive and negative experiences of the model and self-events that were correctly recalled by young and older adults.

**Table 3:** Mean proportion of correct recall of future positive and negative autobiographical events (*SDs* in parentheses) of both the model and own experiences in young and older adults.

Participants	Young		Older	
Model age	Young	Older	Young	Older
**Model events recall**				
Positive	0.62 (0.14)	0.54 (0.16)	0.40 (0.17)	0.40 (0.18)
Negative	0.48 (0.20)	0.44 (0.20)	0.24 (0.14)	0.32 (0.20)
**Self events recall**				
Positive	0.65 (0.18)	0.65 (0.15)	0.52 (0.19)	0.57 (0.19)
Negative	0.55 (0.21)	0.56 (0.20)	0.35 (0.19)	0.36 (0.24)

In order to analyze the number of experiences correctly recalled, a 2 (age group: young or older) × 2 (model age: young or older) × 2 (emotional valence: positive and negative) × 2 (experiences recalled: model and self) ANOVA was conducted. The factors age group, *F*(1,132) = 62.81, *p* < 0.001, $\eta_{p}^{2}$ = 0.32, experiences recalled (model and self), *F*(1,132) = 67.32, *p* < 0.001, $\eta_{p}^{2}$ = 0.34, and emotional valence, *F*(1,132) = 55.86, *p* < 0.001, $\eta_{p}^{2}$ = 0.30, were all significant. As in the production task, young participants (*M* = 0.56) remembered a greater proportion of experiences than the older participants (*M* = 0.40). Relative to the young and old participants, globally, own imagined experiences (*M* = 0.53) were better remembered than the experiences of the model (*M* = 0.43) and more positive experiences (*M* = 0.55) than negative experiences (*M* = 0.41) were recalled. The model age factor, *F*(1,132) = 0.04, *p* = 0.85, $\eta_{p}^{2}$ = 0.01, and the interaction between age group × model age, *F*(1,132) = 1.87, *p* = 0.17, $\eta_{p}^{2}$ = 0.01, were not significant. Thus, having a model with experiences provided by people of the same or different age, young or old, had no impact on recall.

**FIGURE 1:**
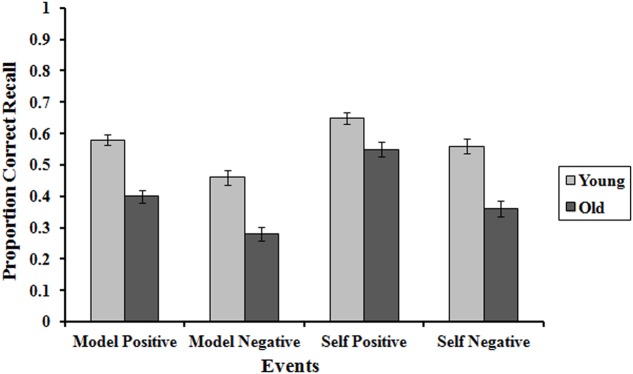
Proportion of correct recall of the positive and negative future events of both the model and own experiences in young and older adults. Error bars represent standard errors.

Only the interaction between age group x experiences recalled (model or self) x valence, *F*(1,132) = 5.40, *p* = 0.022, $\eta_{p}^{2}$ = 0.04, was significant. *Post hoc* comparisons were performed using the Bonferroni test. We found (**Figure 1**) that the differences between young and old were smaller for the recall of their own positive experiences (0.65 -0.56 = 0.09) in comparison with their own negative experiences (0.56 -0.36 = 0.20), positive model experiences (0.58 -0.40 = 0.18) and negative experiences (0.46 -0.28 = 0.18), with *p* < 0.05 in all comparisons. Thus, as in the production task, in correct recall we also find an age-based positivity effect. In addition, in older people the differences between the recall of positive and negative experiences were greater when the experiences were their own (0.55 -0.35 = 0.20) as opposed to the model experiences (0.40 -0.28 = 0.12), *t*(67) = 2.03, *p* < 0.05, *d* = 0.26, whereas in the young there were no significant differences between recall of positive and negative experiences between recall of model experiences (0.58 -0.46 = 0.12) and own experiences (0.65 -0.56 = 0.09), *t*(67) = -1.10, *p* = 0.27, *d* = 0.12. Therefore, not only do the participants remember their own experiences better than those of the model, the older participants are more positively biased than the younger participants, but only when asked to recall their own experiences.

### Errors

Three types of errors were taken into account for the recall task. Commission errors were analyzed, in which the participants recalled experiences or content that was neither in the model nor in the experiences they had produced themselves. Source errors, where they mistook in the recall task the model and their own experiences (e.g., remembering as own experience to travel to Paris, when it was only a model experience) or attributed subjects and actions incorrectly (e.g., if they had produced to discuss with their sister-in-law, remembering to discuss with their brothers), and errors of valence change, which consisted of remembering negative experiences as being positive (e.g., a negative event such as not being able to finish the course being remembered as positive, i.e., finishing the course) or remembering positive experiences as having negative valence (e.g., the positive event of buying a new house being remembered negatively, such as not being able to buy a new house). The types of errors were always independent of one another.

The young participants incorrectly recalled an average of 0.85 experiences (*SD* = 0.84, range 0–4) and the older participants a mean of 0.92 experiences (*SD* = 1.09, range 0–7). The mean number of errors was less than 1 in the recall of self events (*M* = 0.82, *SD* = 0.90), *t*(135) = -2.39, *p* = 0.018, *d* = 0.21, and there were no significant differences in the recall of model experiences (*M* = 0.95, *SD* = 1.05), *t*(135) = -0.57, *p* = 0.57, *d* = 0.05. **Table 4** presents the results of errors in the recall of the positive and negative experiences of both the model and self events in the young and older adults.

**Table 4:** Mean number of errors in the recall of future positive and negative events from the model and own events in young and older adults (*SDs* in parentheses).

Participants	Young		Older	
Experiences	Positive	Negative	Positive	Negative
**Model events recall**				
Commission errors	0.13 (0.38)	0.15 (0.43)	0.07 (0.26)	0.06 (0.24)
Source errors	0.07 (0.26)	0.28 (0.57)	0.07 (0.26)	0.10 (0.31)
Valence change	0.03 (0.17)	0.32 (0.58)	0.06 (0.24)	0.56 (1.07)
**Self events recall**				
Commission errors	0.06 (0.29)	0.09 (0.29)	0.03 (0.17)	0.06 (0.24)
Source errors	0.09 (0.29)	0.09 (0.29)	0.03 (0.17)	0.07 (0.26)
Valence change	0.03 (0.17)	0.35 (0.54)	0.07 (0.26)	0.66 (0.89)

In order to analyze the errors, we conducted a 2 (age group: young or older) × 2 (model age: young or older) × 2 (emotional valence: positive and negative) × 2 (experiences recalled: model and self) × 3 (type of errors: commission, source, and valence change) ANOVA. The factors age group, *F*(1,132) = 0.35, *p* = 0.56, $\eta_{p}^{2}$ = 0.003, model age (young, older), *F*(1,132) = 0.03, *p* = 0.87, $\eta_{p}^{2}$ = 0.001, and experiences recalled (model, self), *F*(1,132) = 2.09, *p* = 0.15, $\eta_{p}^{2}$ = 0.02, were not significant, whereas there were significant effects of the emotional valence, *F*(1,132) = 53.15, *p* < 0.001, $\eta_{p}^{2}$ = 0.29, and in the type of errors, *F*(2,132) = 23.80, *p* < 0.001, $\eta_{p}^{2}$ = 0.15. There were more errors when recalling negative experiences (*M* = 0.70) than positive experiences (*M* = 0.18). The *post hoc* comparisons using the Bonferroni test showed that there were more valence change errors (*M* = 0.52) than source errors (*M* = 0.20) and commission errors (*M* = 0.16), with *p* < 0.001 values in both comparisons. There were no significant differences between source errors and commission errors.

**FIGURE 2:**
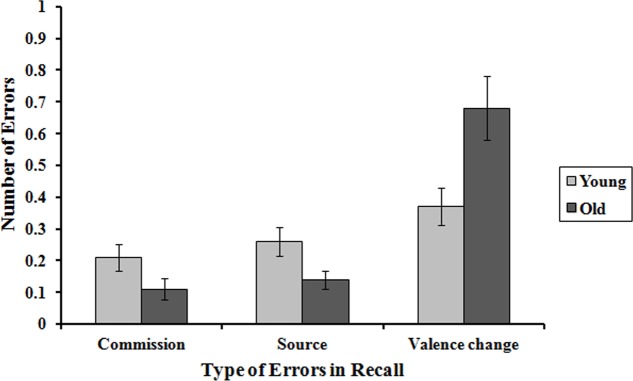
Mean number of errors in the recall of future events in young and old adults. Error bars represent standard errors.

**FIGURE 3:**
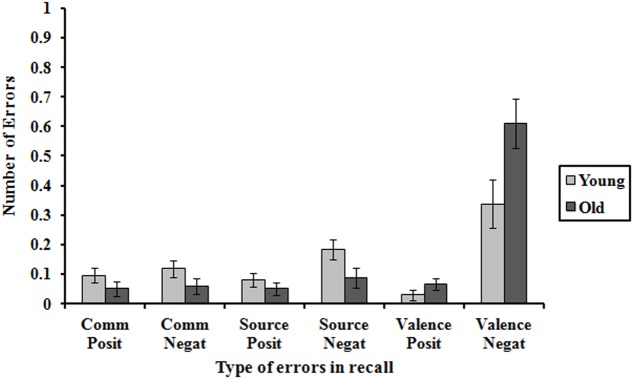
Mean number of commission errors, source errors, and change of valence errors in the recall of positive and negative future events in young and older adults. Error bars represent standard errors.

The interactions age group × error type, *F*(2,132) = 9.12, *p* < 0.001, $\eta_{p}^{2}$ = 0.07, and valence × error type, *F*(2,132) = 27.70, *p* < 0.001, $\eta_{p}^{2}$ = 0.17, were significant. The younger participants showed more source errors than the older participants (0.26 > 0.14, *p* < 0.05), whilst the older participants made more valence change errors than the younger participants (0.68 > 0.37, *p* < .01). There were no significant differences in commission errors between the young and old participants (0.21, 0.11, *p* = 0.06) (**Figure 2**). There were more source errors in the recall of negative experiences than the positive ones (0.14 > 0.07, *p* < 0.05) and valence changes (0.47 > 0.05, *p* < 0.001), but there were no significant differences between positive and negative experiences in terms of commission errors (0.9, 0.7, *p* > 0.05). Also significant was the interaction group × valence × errors, *F*(2,132) = 3.61, *p* < 0.05, $\eta_{p}^{2}$ = 0.03 (**Figure 3**). The differences in errors between young and old were restricted to negative experiences. Only in the recall of negative experiences did the younger participants show more source errors than the older group (0.18 > 0.09, *p* < 0.05), whilst the older participants made more valence change errors than the younger participants (0.61 > 0.34, *p* < 0.05). The change in the value of negative to positive experiences was twice as great in the elderly as in the young. Therefore, the errors also reveal an age-based positivity effect.

Also significant was the interaction model age × valence × experiences recalled (model or self) × errors, *F*(3,132) = 3.21, *p* = 0.023, $\eta_{p}^{2}$ = 0.03. There were more source errors in the recall of the experiences of the model than in the own experiences (0.22 > 0.06, *p* < 0.01) and more changes of negative to positive valence in the recall of the own experiences than in the recall of the model experiences (0.56 > 0.34, *p* < 0.05). The source errors in the recall of the model experiences show that the encoding of the model was more superficial than that of the own experiences. However, valence errors show that age-based positivity effects are applied more frequently to the own experiences than to those of the model. Thus, whilst self-referencing favors memory it also fosters biases of emotional self-adaptation toward positivity.

## Discussion

The central objective of this study was to examine age-based positivity effects in the recall of emotional future events. The participants, young and older adults, were presented with a model of future experiences provided by a previous study in young and old adults and had to generate their own future experiences from positive and negative retrieval cues. Although the participants knew that it was a memory experiment, they were never told that they had to study the experiences or that they would have to remember them later. That is, they had no restrictions or complex instructions to follow. It has been observed that the positivity effect is more pronounced in studies that do not constrain cognitive processing and allow the participants’ preferences to appear more spontaneously (Reed et al., 2014). Therefore, whilst incidental processing can reduce the level of recall (Schlagman et al., 2009), these are the ideal conditions under which the positivity effect, the interaction between age (young vs. old) and emotional valence (positive vs. negative), can naturally emerge. As expected, both our young and older participants remembered more positive than negative events, showing a clear optimism bias about the future. In addition, age-based positivity effects were evident in the older participants, since on both the production and recall tasks the elderly group was relatively more resistant to the recall of negative events (Charles et al., 2003). Further, when examining errors the older group transformed twice as many negative future events into positive experiences. Our results are consistent with the Socio-emotional Selectivity Theory (SST; Carstensen, 2006), which emphasizes the importance of prioritizing information related those aspects that generate wellbeing and balance. Although studies are needed to determine the role of inhibitory mechanisms in the positivity effect, findings with young adults in autobiographical memory suggest that people tend to reduce, suppress or block the accessibility of negative thoughts and events (Giebl et al., 2016; García-Bajos and Migueles, 2017).

An interesting aspect of the present study is that it compares young and old participants that share certain characteristics. In particular, all participants, young and old, were university students, a characteristic that homogenizes variables such as culture or socioeconomic status. In this experiment, despite the possible diversity in vital and generational experiences, there were no differences between young and old in terms of years of formal education. The age limit of the older participants was capped at 75 years to prevent high levels of cognitive impairment. Young and old people with serious medical or psychological problems, such as cancer or depression, were also excluded. Older people tend to perceive their health to be worse than young people (Pinquart, 2001) but, after applying these selection criteria, there were no significant differences between the young and older participants in their overall health in this study. Nonetheless, when cognitive abilities are compared, the common limitations associated with aging appear. In the present study three cognitive aspects were evaluated. Young adults showed greater semantic fluency than older adults in a production task of exemplar categories. The older participants had higher scores in verbal comprehension than the young adults when tested on a synonyms task. And older people were at a disadvantage compared with young people when tested on a digit-symbol coding task, which involves greater cognitive abilities, working memory, and processing speed. These results are in agreement with the findings of other studies on cognitive aging, which significantly affects the speed of processing and working memory, but not world knowledge (Park et al., 2002).

In this study we analyzed the positivity effect both in the production task and in the correct and incorrect responses on the recall task. In all three measures we found a preference for the positive contents to the detriment of the most negative contents. As in many studies on episodic future thinking (e.g., Kwan et al., 2010; Gallo et al., 2011), a cueing technique was used to obtain future autobiographical events. Although several studies point out that future events produced in response to experimentally provided cues are of a different nature compared with self-generated future events (Neroni et al., 2016), the cues used in this experiment came from a previous normative study, providing cues to generate relevant emotional facts for each age group. This procedure allowed us to examine the correct recall and the nature of the errors of personal emotional events and those of others of the same age or a different age. In the production task, although the size of the effect was small, the younger participants produced more future events than the elderly group. This result, however, was restricted to negative experiences, because there were no significant differences between the young and old participants in the production of positive events. Therefore, older adults produced fewer negative events than the younger group, which indicates a positivity effect on the task of producing future events in the elderly. With increasing age, motivational priorities change, and a preference for positive information over negative information emerges (Carstensen and Mikels, 2005; Mather and Carstensen, 2005; Schryer and Ross, 2014). Older people have greater accessibility to positive future thoughts, and give priority to thoughts that generate satisfaction or emotional balance. In fact, during the production phase the older participants expressed that they had difficulty imagining negative future events and that they preferred to think positively.

In the recall task, as in the production task, young adults remembered, regardless of emotional valence, more future experiences than the older participants. One of the more consistent findings in the cognitive aging literature is that, compared with younger adults, older adults provide less information and are less accurate. The deterioration in performance in episodic memory tasks, as used in the present study, has been observed in a range of situations including word lists, sentences, fragments of prose, faces, drawings, photographs, or daily life situations (see Salthouse, 1991; Bäckman et al., 2000; and Park et al., 2002, for reviews). In addition, the limitations related to aging are accentuated on tasks of free recall where there are no external cues, and which require self-initiation and the use of retrieval strategies (Craik, 2005). Our results support the notion of a negative effect of aging on the recall of imagined future events.

What was the impact of remembering one’s own or others’ experiences? Participants recalled more self events than those of the model, regardless of whether the model was young psychology students or older students. As shown in previous studies, both young and older adults can benefit from self-referenced items (Gutchess et al., 2007; Lalanne et al., 2013). The task of generating one’s own experiences involves a deeper level of processing than simply reading the experiences provided by other people, and it is known that more elaborate processing leads to better performance on memory tasks (Craik and Lockhart, 1972; Craik, 2002). Therefore, the processing of the model could be more superficial, based on the simple reading of the experiences without elaboration, whereas producing own personal experiences entails more in-depth processing. Both young and older adults can benefit of depth of processing (Lalanne et al., 2013). In addition, the experiences generated by the participants themselves were aspects of personal relevance, and represented plausible and highly significant events that could occur in the near future (D’Argembeau et al., 2012), and events that are relevant to the self are better remembered than the experiences of other people (Gutchess et al., 2007; Carson et al., 2016).

The self is a meaningful construct that is linked to motivational and social aspects that become increasingly relevant with age (Gutchess et al., 2007). Encoding information with reference to the self can be a natural and familiar strategy for the elderly, which can help to reduce the cognitive processing burden and minimize differences when compared with the performance of young people (Castel, 2005; Gutchess et al., 2007). Thus, relating information to oneself could be an effective encoding strategy that helps to process the information in a meaningful, elaborate, and organized way (see Klein and Loftus, 1988; Leshikar et al., 2015). Contrary to the idea that self-reference effect can be more marginal for older than younger adults (Lalanne et al., 2013), the opposite pattern of results was observed in the present experiment. Further, and contrary to the inconsistent results with a positivity effect in aging found by Leshikar et al. (2015), that self-referencing increased recollection memory for positive items in both young and older adults, a significant positivity effect was evident in the present experiment in the recall of own imagined future events. In fact, the impact of the self was more relevant in older people, particularly when dealing with the positive social-emotional information to which they are motivated to pay special attention. These ideas are supported by the fact that the differences between young and old were less evident in the recall of positive experiences than negative experiences, both for those experiences related to the self and those of the model. Another result consistent with the relevance of self-reference processing and positivity effect is that in older people the differences between the recall of positive and negative experiences were greater for the recall of self future events than those of the model, while in the young people these differences did not appear. Working with the experiences of other people of the same generation or a different generation, whilst it could also have triggered processes of self-referencing and identification as individuals attempt to understand the mental state of others (Hess et al., 2013), was not a relevant variable, possibly because they did not perceive the experiences of the model as being relevant or as pertaining to themselves, or because older adults did not identify with the model of their chronological age (e.g., Rubin and Berntsen, 2006).

Relatively few studies have documented the types of errors made in recall in young and older adults. Compared with younger adults, older adults are impaired in their ability to accurately recall perceptual and conceptual based source information but age differences are reduced or even eliminated when participants process emotional or affective information (May et al., 2005). In this experiment three types of errors were taken into account. First, we examined commission errors, which are due to own elaborations, deductions or reconstructive processes based on typical aspects, stereotypes, or knowledge schemas for such events. Second, we considered source attribution errors, which are due to erroneous subject/action exchanges, and which may depend on the level of processing. For this reason, the attribution errors were more likely in the recall of the model than in the recall of one’s own imagined future events. Age-related differences in source errors were restricted to the recall of negative events and, contrary to predictions of age-related decline in source accuracy based on recognition tasks (see May et al., 2005; Gallo et al., 2011), young adults had more source errors than older adults. This result may be derived from the worse recall of negative events in older adults. Third, we looked at the valence change errors. The change from negative to positive valence in recall was more pronounced in the recall of one’s own experiences than those of the model. This effect is due to a tendency toward self-adaptation that biases our perspective toward positive events. We are motivated, particularly the elderly, to process information that generates emotional satisfaction and we have a desire to attain a level of emotional wellbeing (Carstensen, 2006). Transforming the negative, which disturbs or worries us, into something positive is an example of this bias. Our results are in line with Fernandes et al. (2008) study. They measured memory for words, pictures and autobiographical material in young and older adults and on all three recall tasks older adults erroneously recalled more positive than false negative memories, showing that older reconstruct the past to accentuate the positive. Even so, consistent with the idea that free recall tasks generate few errors, the average number of errors was less than one per participant in this experiment, as occurs, for instance, in the recall of emotional eyewitness events (e.g., García-Bajos et al., 2012). Nonetheless, the information they provide is very significant because it illustrates the processes involved and the biases derived from constructing and remembering positive and negative future events.

In our results we found that there were more valence change errors than source or commission errors. The positive to negative valence change represented only 9% of all valence changes, while the change from negative to positive events was 91%. Therefore, the most characteristic error was the tendency to favor positive content over negative content in the recall of future events. There were more source errors in negative events than positive events for the model experiences; this difference, however, was less evident for the own experiences, which could indicate that negative events involving others could receive a more superficial and less detailed level of processing, and therefore the connection between subject and action is lost more easily. Contrary to what happens on recognition tasks that generate high proportions of false alarms, commission errors were infrequent in recall and there were no significant differences between young and older participants or between positive and negative events in this regard.

With respect to age differences, the most important errors were the changes in valence from negative to positive events, which were twice as frequent in the older participants in comparison with the younger ones. Therefore, in this experiment, as in the production and the correct recollection of future events, examining the errors also reveals a strong age-based positivity effect in the elderly. One striking feature is that positivity effects are more often applied to one’s own experiences than to those of the model. That is, self-referencing favors memory (Klein and Loftus, 1988; Gutchess et al., 2007), but also encourages biases to avoid the negative and to have a vision of emotional self-adaptation that favors positivity.

## Conclusion

The results of this study show a consistent optimism bias in the memory of future events, that is, we have a greater tendency to remember positive experiences than negative experiences. Thus, imagining the future may have synergistic value for our everyday emotional state. Compared with younger people, older people show a positivity effect, a greater preference for positive content that brings them wellbeing, balance and personal satisfaction. But our data also show another adaptive characteristic of memory, i.e., the rejection of the emotionally negative. People tend not to think about the negative things that may come to them in the future and that generate anguish, fear, or sadness. Our data suggest that older people are particularly prone to avoid negative experiences of the mind and to even transform them into more emotionally pleasant ideas. Because of its social relevance and potential applicability to clinical settings, it will be a challenge for future research to examine which variables encourage positivity when imagining the future, along with the real impact of this effect on everyday life.

## Author Contributions

All the authors contributed to the project of this research. MM and EG-B conceived, designed and prepared the materials for the experiment. MM and AA performed the experiment and collected the data. EG-B and MM scored the tasks and EG-B analyzed the data. EG-B and MM wrote the manuscript. All the authors revised critically the paper for important intellectual content, approved the manuscript for publication, and agreed to be accountable for all aspects of the work in ensuring that questions related to the accuracy or integrity of any part of the work are appropriately investigated and resolved.

## Conflict of Interest Statement

The authors declare that the research was conducted in the absence of any commercial or financial relationships that could be construed as a potential conflict of interest.
